# Caboxamycin Inhibits Heart Inflammation in a Coxsackievirus B3-Induced Myocarditis Mouse Model

**DOI:** 10.3390/v16050677

**Published:** 2024-04-25

**Authors:** Hong-Gi Kim, Prima F. Hillman, You-Jeung Lee, Ha-Eun Jeon, Byung-Kwan Lim, Sang-Jip Nam

**Affiliations:** 1 Department of Biomedical Science, Jungwon University, Goesan-gun 28024, Chungbuk, Republic of Korea; ghdrl368@naver.com (H.-G.K.); wjs20007@gmail.com (H.-E.J.); 2 Department of Chemistry and Nanoscience, Ewha Womans University, Seoul 03760, Republic of Korea; primafitriah@gmail.com; 3 Division of Cardiology, Samsung Medical Center, 50 Irwon Dong, Gangnam-gu, Seoul 06351, Republic of Korea; eelulee@daum.net

**Keywords:** *Streptomyces* sp. SC0774, Coxsackievirus B3, myocarditis, caboxamycin

## Abstract

Coxsackievirus B3 (CVB3) is a positive single-strand RNA genome virus which belongs to the enterovirus genus in the picornavirus family, like poliovirus. It is one of the most prevalent pathogens that cause myocarditis and pancreatitis in humans. However, a suitable therapeutic medication and vaccination have yet to be discovered. Caboxamycin, a benzoxazole antibiotic isolated from the culture broth of the marine strain *Streptomyces* sp., SC0774, showed an antiviral effect in CVB3-infected HeLa cells and a CVB3-induced myocarditis mouse model. Caboxamycin substantially decreased CVB3 VP1 production and cleavage of translation factor eIF4G1 from CVB3 infection. Virus-positive and -negative strand RNA was dramatically reduced by caboxamycin treatment. In addition, the cleavage of the pro-apoptotic molecules BAD, BAX, and caspase3 was significantly inhibited by caboxamycin treatment. In animal experiments, the survival rate of mice was improved following caboxamycin treatment. Moreover, caboxamycin treatment significantly decreased myocardial damage and inflammatory cell infiltration. Our study showed that caboxamycin dramatically suppressed cardiac inflammation and mouse death. This result suggests that caboxamycin may be suitable as a potential antiviral drug for CVB3.

## 1. Introduction

Enteroviruses can trigger severe arrhythmias and abrupt cardiac death as well as the development of chronic myocarditis and heart failure [1,2,3,4]. Also, in children, enteroviruses are the causes of central nervous system (CNS) infections, such as infant meningitis and encephalitis, which have non-specific symptoms and may include acute neurologic complications and complex seizures [5,6,7]. Hand, foot, and mouth disease caused by enterovirus71 (EV71) can cause aseptic meningitis and brain stem encephalitis with respiratory failure and is associated with high mortality [8,9]. Therefore, enteroviruses are considered very important emerging viral agents. Among them, Coxsackievirus B3 (CVB3) is one of the most common causes of myocarditis [10]. CVB3 protease 2A and 3C cleave viral polyproteins during virus replication. These enzymes destroy a variety of host proteins required for cell structure maintenance, protein translation, transcription, and cell signaling. Transgenic mice with cardiac-specific localization of protease 2A develop severe cardiac dysfunction and dilated cardiomyopathy. These findings suggest that inhibiting protease 2A activity might be an effective method to treat CVB3 infection [11,12]. Previous studies reported the antiviral activity of a few possible antiviral drugs in an experimental CVB3 acute murine myocarditis model in the Balb/C strain [13]. For instance, IL-1 receptor antagonist (IL-1 Ra) trap, Coxsackievirus and Adenovirus receptor (CAR) trap gene therapy, and 3C protease inhibitor (3CPI) were investigated in the Balb/C strain as a model of acute severe myocarditis. In addition, the antiviral effects of water-insoluble CVB3 3CPI in 100% dimethyl sulfoxide (DMSO), which was supplied by implanted micro-osmotic pumps in a subcutaneous region in a Balb/C mouse model, has also been reported recently. The result demonstrated that 3CPI effectively suppressed CVB3-induced myocardial damage and mortality [14]. However, there are many limitations to developing therapeutic drugs for clinical application.

Viruses cause a variety of human pathogeneses, including cancer, and play an essential role in human disease. Although improvements in vaccination and drug development have been made, numerous viruses lack preventive vaccines and effective antiviral medications, which are frequently affected by viral escape mutants. As a result, discovering new antiviral drugs is crucial. Herbal medicines and natural products are a rich resource for discovering new antiviral drugs [15]. Our study shows that the natural extract compound Fructose Amomi extract strongly inhibits the activity of the extracellular signal-regulated kinase (ERK) pathway and protein kinase B (AKT), both of which are major signaling pathways that regulate CVB3 replication and cellular toxicity [16,17,18]. This extract strongly attenuated virus replication in HeLa cells. In particular, Fructose Amomi extract protected the mouse heart against damage and improved mouse survival in a CVB3-induced myocarditis mouse model [19].

**Figure 1:**
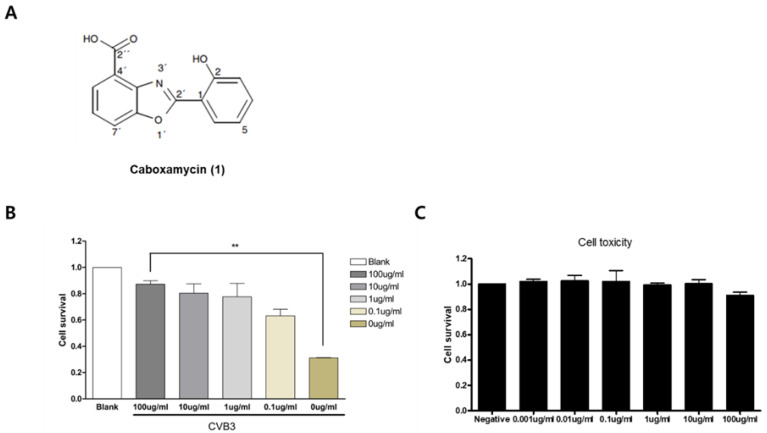
Identification of compound 1. (**A**) A new antiviral drug was isolated and purified from ocean microorganisms. The structure of caboxamycin (**1**). HeLa cells were treated with serially diluted caboxamycin (1) (100 to 0.1 μg/mL) with or without CVB3 infection (0.1 MOI) at the same time for 18 h. Then, a microplate reader measured cell survival. (**B**) Caboxamycin significantly improved virus-infected cell survival. (**C**) Cell toxicity of caboxamycin was observed by inducing HeLa cell. The purified drug did not show cytotoxicity. All data are presented as the mean ± standard deviation (SD) from repeated experiments. ** *p* < 0.01 in two-tailed Student’s *t*-test.

Marine-derived actinomycetes have been considered an essential source of various biological functions, including antiviral, anti-tumor, antimicrobial, and anticancer [20,21]. Caboxamycin was first isolated from the extracts of the marine *Streptomyces* sp. NTK 937. The compound possesses a benzoxazole scaffold as the core center, as shown in Figure 1, and was reported to exhibit antibiotic activity against Bacillus subtilis as well as against the yeast Candida glabrate [22,23]. We used the culture broth of *Streptomyces* sp. strain SC0774, isolated from marine sediment in the Antarctic, to generate caboxamycin (**1**) (Figure 1A). In this study, we describe the isolation of caboxamycin (**1**) and demonstrate the antiviral activity of caboxamycin (**1**) against CVB3.

In this study, we observed that caboxamycin (**1**) significantly suppressed cardiac myocyte apoptosis and effectively inhibited the proliferation of CVB3 in vitro and in vivo. This result suggests that caboxamycin (**1**) could be applied in the development of a new antiviral drug against CVB3-induced myocarditis.

## 2. Materials and Methods

### 2.1. General Experimental Procedure

Low-resolution LC/MS measurements were carried out using an Agilent Technologies 1260 quadrupole (Agilent Technologies, Santa Clara, CA, USA) and Waters Micromass-ZQ 2000 MS system (Waters Corp, East Lyme, CT, USA) using a reversed-phase column (Phenomenex Luna C-18 (2), 50 mm × 4.6 mm, 5 µm, 100 Å) at a flow rate of 1.0 mL/min at the National Research Facilities and Equipment Center (NanoBioEnergy Materials Center) at Ewha Womans University. A solvent signal as an internal standard on Varian Inova spectrometers (Bruker, Billerica, MA, USA) was used to collect 1H and 2D NMR spectra at 400 MHz in CDCl3. Also using the Varian Inova spectrometer, the 13C NMR spectrum was acquired at 100 MHz. Open-column chromatography was performed on C-18 resin (40–63 μm, ZEO prep 90) with a gradient solvent of water (H_2_O) and methanol (MeOH). The fractions acquired from open-column chromatography were subsequently purified by reverse-phase high-performance liquid chromatography (HPLC) using a Phenomenex Luna C-18 (2), 100 Å, 250 nm × 10 mm, 5 μm column with a mixture of acetonitrile (CH_3_CN) and H_2_O at a flow rate of 2.0 mL min^−1^.

### 2.2. Collection and Phylogenetic Analysis of Strain SC0774

The marine-derived actinomycete strain SC0774 was obtained from a marine sediment sample acquired in the Antarctic. Based on NCBI blast analysis of partial 16S rRNA, the strain SC0774 was identified as *Streptomyces* sp., with 100% similarity to *Streptomyces griseolus*. The gene sequence data are available from Genebank (deposit MW132412.1).

### 2.3. Fermentation, Extraction, and Isolation

The strain SC0774 was cultivated in 80 L of 2.5 L Ultra Yield Flasks, containing 1 L of SYP SW medium (10 g/L of soluble starch, 2 g/L of yeast, 4 g/L of peptone, and 139 g/L of sea salt in 1 L of distilled water) in each flask at 27 °C and was shaken at 120 rpm for 7 days. After a culture period of 7 days, the culture medium was extracted with ethyl acetate (EtOAc), obtaining a total of 80 L of extract, which was concentrated in a rotary vacuum evaporator to obtain 4.0 g of crude extract. The crude extract was fractionated into 8 fractions using reverse-phase C18 flash chromatography with a stepwise gradient elution of 80% of H_2_O in MeOH to 100% MeOH. The sixth fraction, 80% MeOH in H_2_O, was purified by reverse-phase HPLC (Phenomenex Luna C-18 (2), 250 × 100 mm, 2.0 mL/min, 5 μm, 100 Å, UV = 210 nm) using isocratic condition with 45% aqueous CH_3_CN to yield 4.9 mg of compound **1**.

### 2.4. Viruses and Cells

Coxsackievirus B3 (CVB3) was generated from an infectious cDNA copy of the cardiotropic CVB3-H3 and was amplified in HeLa cells. Virus-infected HeLa cells and tissue virus titer were defined using plaque forming unit (PFU) assay [24]. The HeLa cells were cultured in Dulbecco’s Modified Eagle’s medium (DMEM, Welgene, Inc., Gyeongsan-si, Republic of Korea) enriched with 10% fetal bovine serum (FBS) and 1% penicillin–streptomycin solution (Welgene, Inc., Gyeongsan-si, Republic of Korea) solution at 37 °C.

### 2.5. Screening and Optimization of Antiviral Compounds

Antiviral activity was observed following previous reports. Briefly, HeLa cells cultured on a 96-well plate were infected with 2 × 10^4^ PFU/mL of CVB3-H3 (0.1 MOI) and co-administrated with individual compounds, which were serially diluted from 100 μg/mL to 1 ng/mL. At 16 h post-infection, cell survival was evaluated with an additional 8 μL of Cell Counting Kit 8 (CCK-8; Dojindo Molecular Technologies, Inc., Rockville, MD, USA) reagent.

### 2.6. Myocarditis Mouse Model and Drug Administration

The protocols used in this study corresponded to the Guide for the Care and Use of Laboratory Animals published by the US National Institutes of Health (NIH Publication No. 85–23, revised 1996). On day 0, six-week-old male Balb/C mice were infected with 2 × 10^5^ plaque-forming units (PFU) of CVB3-H3 by intraperitoneal injection. The mice were euthanized via cervical dislocation and sera and various tissues (heart, liver, spleen, and pancreas) were acquired on days 7 and 14 [25]. Caboxamycin (100 μg/mL in 100 μL saline or saline alone) was administered intraperitoneally from day 0 post-infection for five consecutive days (CVB3 + caboxamycin group (CVB3 + Cabo), *n* = 10; CVB3 group (CVB3); *n* = 10). The hearts, livers, and pancreas of the mice were taken out and evaluated on days 3, 7, and 12 post-infection (p.i). Every procedure was evaluated and authorized by the Institutional Animal Care and Use Committee of Samsung Biomedical Research Institute (SBRI, #20221226001).

### 2.7. Quantitative Real-Time PCR

RNA was obtained from virus-infected mouse hearts. Total RNA was obtained using TRIzol^®^ reagent (ThermoFisher Scientific, Cambridge, MA, USA) according to the manufacturer’s protocol. Complementary DNA (cDNA) was synthesized using 1 μg RNA. Real-time PCR was used to analyze quantitative RNA using the SYBR Green system (Applied Biosystems, Waltham, MA, USA). Real-time PCR conditions were 95 °C for 10 min, then 40 cycles at 95 °C (30 s) and 60 °C (60 s), and then a denaturation curve. The primer sequences are shown in Table 1.

### 2.8. Immunoblot Analysis

HeLa cell protein was isolated using 1X PBS lysis buffer (1X PBS, 0.5 mM EDTA, Triton X-100, and protease inhibitor mixture) and mixed with sample buffer. The protein was separated on a 10% SDS-PAGE system and then carried to nitrocellulose membranes. The membranes were probed with the anti-CVB3 VP1 antibody (ThermoFisher Scientific, Cambridge, MA, USA), eIF4G1, BAX, BAD, phosphor ERK, total ERK, cleaved caspase3, and GAPDH antibodies (Cell Signaling, Danvers, MA, USA) and refrigerated for 18 h at 4 °C. Antibody binding was detected using the ECL system (Intron Biotech, Inc., Seongnam-si, Republic of Korea) and the results were quantified using NIH-ImageJ1.45s software (Wayne Rasband, MA, USA).

### 2.9. Histologic Findings

The hearts and pancreases were secured in 10% normal saline formalin, and hematoxylin and eosin (H&E) stain was conducted using paraffin-embedded sections as previously described [26]. The staining tissue was identified under a light microscope. Tissue images were captured and analyzed using a light microscope (Olympus Co., San Jose, CA, USA). The inflammatory cell infiltration area (%) was quantified using NIH-ImageJ1.45s software.

### 2.10. Statistical Analysis

Statistical analysis was performed using Prism 9 software (GraphPad Software, San Diego, CA, USA) and provided as means ± standard deviations (SDs). The statistical significance of the two groups was determined using a Student’s *t*-test. A two-way ANOVA was used to analyze multiple time-point experiments. * *p* < 0.05, ** *p* < 0.01, *** *p* < 0.001 indicated significance.

## 3. Results

### 3.1. Compound ***1*** Characterization

Compound **1** was obtained as a white powder, and its pseudomolecular ion peak at *m*/*z* = 256.23 [M + H]^+^ was revealed in LRMS spectroscopic data. Compound **1** was identified as caboxamycin (Figure 1A) by comparing its NMR data to the literature. Caboxamycin (**1**) was first isolated from the extracts of the marine *Streptomyces* sp. NTK 937. Compound **1** possessed a benzoxazole scaffold as the core center, as shown in Figure 1, and was reported to exhibit antibiotic activity [22,23].

### 3.2. Caboxamycin Strongly Inhibited Coxsackievirus B3 Replication

**Figure 2:**
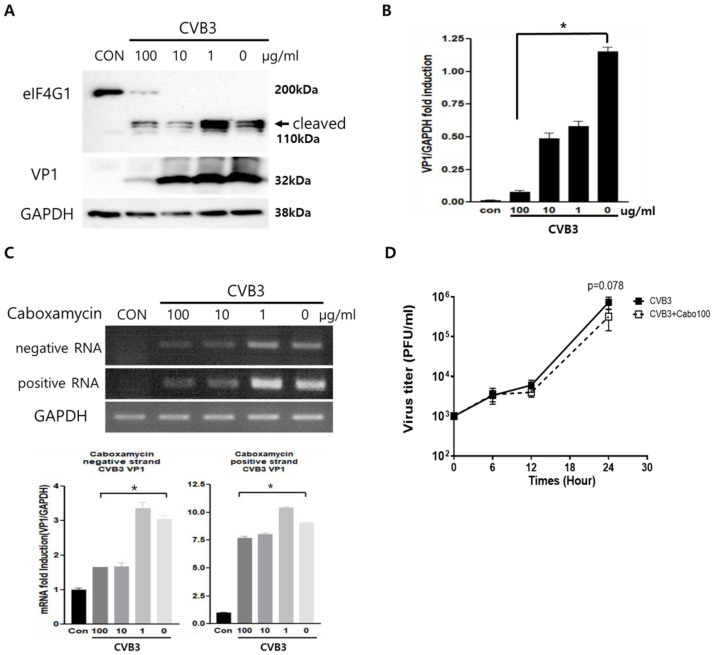
The effect of caboxamycin (1) inhibits EV71 replication. (**A**,**B**) HeLa cells infected by CVB3 were treated with serially diluted caboxamycin (1); then, proteins and RNA were extracted at 16 h pi. The extracted protein was used for Western blot analysis and probed by the antibody eIF4G1, enterovirus-VP1, and GAPDH as a protein loading control. Cleaved eIF4G1 (arrow) and viral capsid protein VP1 decreased under 100 μg/mL caboxamycin (1) treatment. (**C**) The extracted RNA was used for semi-quantitative RT-PCR; the primer pair identified the negative and positive-strand RNA genome of EV71. Western blot and RT-PCR results were quantitated using NIH-ImageJ1.45s software. (**D**) one-step growth curve of CVB3 at 1 h post administration of caboxamycin. All data are presented as mean ± SD from repeat experiments. * *p* < 0.05 in two-tailed Student’s *t*-test.

Caboxamycin’s (**1**) antiviral effect was observed in HeLa cells infected with CVB3. CVB3 was co-administered with 100 to 0.1 μg/mL serial diluted caboxamycin (**1**) for 16 h. Cell survival was then measured with a CCK-8 kit. HeLa cell survival was strongly preserved by caboxamycin (**1**) treatment compared to the untreated group (Figure 1B). In addition, caboxamycin (**1**) showed very weak cell toxicity in HeLa cells (Figure 1C). CVB3 replication was observed by Western blot analysis and semi-quantitative RT-PCR of viral capsid protein (VP1). During virus replication, it produces protease 2A protein to cleave their polyprotein to generate viral particles. This protease 2A breaks down the translation initiation factor eIF4G1. Caboxamycin (**1**) treatment dramatically suppressed eIFG1 cleavage and VP1 viral capsid protein production (Figure 2A,B). Virus replication is initiated with mRNA genome amplification. In particular, negative-strand RNA amplification is the main step in virus replication. Treatment with 100 and 10 μg/mL of caboxamycin (**1**) inhibited positive and negative strand RNA genome amplification (Figure 2C). In addition, HeLa cells were infected with 1 MOI CVB3; then, caboxamycin was administered 1 h post-infection (p.i.). Whole-cell lysates were collected 6, 12, and 24 h after treatment with 100 μg/mL caboxamycin. The cell lysates underwent three freezing–thawing cycles and were then subjected to PFU assay to measure virus titers. CVB3 replication was inhibited post-treatment with caboxamycin. However, the data were not statistically significant (*p* = 0.078). These data suggested that caboxamycin’s antiviral effect mainly acted by inhibiting the virus replication process. Caboxamycin (**1**) has a strong inhibition effect on both genome amplification and viral protein production.

### 3.3. Caboxamycin Treatment Inhibited Apoptosis and Improved the Cell Survival Signaling Pathway

**Figure 3:**
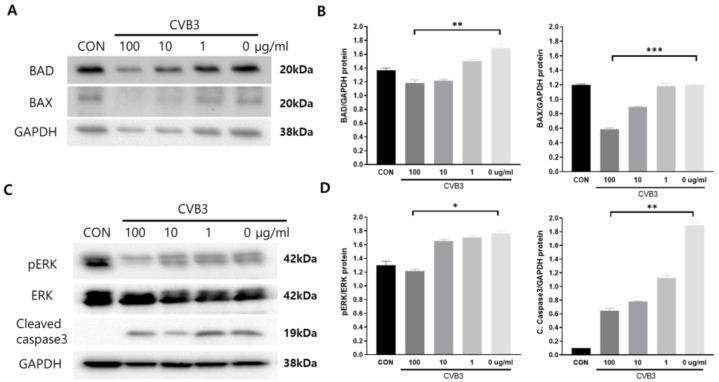
Caboxamycin (**1**) effectively improves cell survival signaling during CVB3 infection. (**A**,**B**) The protein was extracted from HeLa cells after treatment with serially diluted caboxamycin (**1**) during CVB3 infection. Bad and Bax expression levels were quantified using NIH-ImageJ1.45s software. (**C**,**D**) The protein levels of cleaved caspase3, phosphor-ERK, total ERK, and GAPDH expression were observed by immunoblot analysis. Band intensity was measured using NIH-ImageJ1.45s software. All data are presented as mean ± SD from repeat experiments. * *p* < 0.05; ** *p* < 0.01; *** *p* < 0.001 in two-tailed Student’s *t*-test.

Virus infection activates the apoptosis pathway and kills cells. This pathway is regulated by the activation of Bcl-2 family proteins such as pro-apoptotic BAD (Bcl-2 associated Agonist of cell death) and BAX (Bcl-2 Antagonist X). We tested apoptosis inhibition by treating CVB3-infected HeLa cells with caboxamycin (**1**). However, pro-apoptotic BAD and BAX expression levels dramatically increased due to CVB3 infection and were significantly decreased following caboxamycin (**1**) treatment. Moreover, a high dose of caboxamycin (**1**) inhibited pro-apoptosis protein expression (Figure 3A,B). This apoptosis inhibition may be beneficial for antiviral effects on CVB3-infected cells. Moreover, caspase3 cleavage and MAP kinase ERK phosphorylation were significantly decreased following 100 μg/mL caboxamycin (**1**) treatment (Figure 3C,D). These results imply that apoptotic cell signaling was dramatically inhibited by caboxamycin (**1**) treatment. The reduction in apoptotic activity effectively inhibited CVB3 replication.

### 3.4. Caboxamycin Reduced Cardiac Inflammation and Virus Replication in a CVB3-Induced Myocarditis Mouse Model

**Figure 4:**
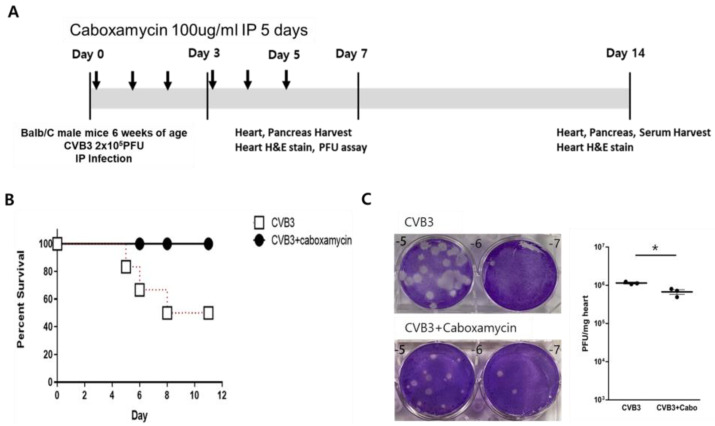
Caboxamycin decreased mouse mortality and heart virus replication in CVB3-induced myocarditis. (**A**) CVB3 was injected intraperitoneally to 6-week-old male Balb/C mice with PBS (CVB3, *n* = 10) or caboxamycin treatment (CVB3 + Cabo, *n* = 10). (**B**) Mouse survival rate was recorded at day 14 pi. (**C**) Hearts were lysed at day 5 pi. PFU assay was performed on tissue lysates to determine tissue virus titers. All data represent mean ± SD. * *p* < 0.05 in two-tailed Student’s *t*-test.

Caboxamycin was administered in a CVB3-induced myocarditis mouse model. The animal experiment was conducted following the experimental design (Figure 4A). Briefly, six-week-old male mice were intraperitoneally infected with 2 × 10^5^ PFU of CVB3 (CVB3, *n* = 10) or with caboxamycin (CVB3 + Cabo, *n* = 10). The survival rate of the mice was preserved in the caboxamycin-treated group compared with the untreated group (CVB3 vs. CVB3 + Cabo; 45 vs. 100%, *p* < 0.08). Heart virus titers were measured by PFU assay. The overall result showed that heart virus proliferation was significantly decreased in the CVB3 + cabo group compared to CVB3 group (CVB3 vs. CVB3 + Cabo: 1,143,000 ± 87,369 vs. 670,233 ± 161,678, * *p* < 0.05) (Figure 4B,C).

**Figure 5:**
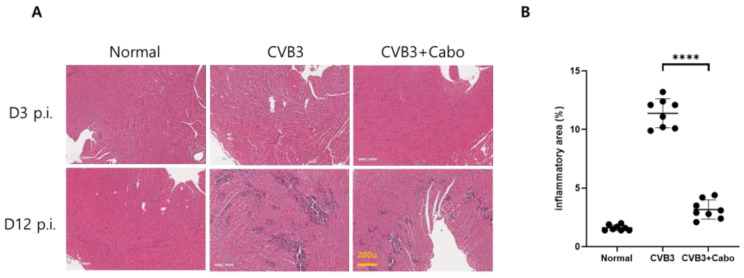
Myocardium inflammation is decreased in caboxamycin-treated mice. (**A**) Heart inflammation in CVB3-infected mice was observed by heart histological findings. Mice were sacrificed on day 3 and day 12 pi, and then the serum and heart tissues were collected. Histological findings in the heart using hematoxylin and eosin (H&E) staining showed inflammatory cell infiltration. (**B**) Quantification of inflammation area (%) in the heart (CVB3 vs. CVB3 + Cabo, n = 3). All data are presented as mean ± SD. **** *p* < 0.0001 in two-tailed Student’s *t*-test.

**Figure 6:**
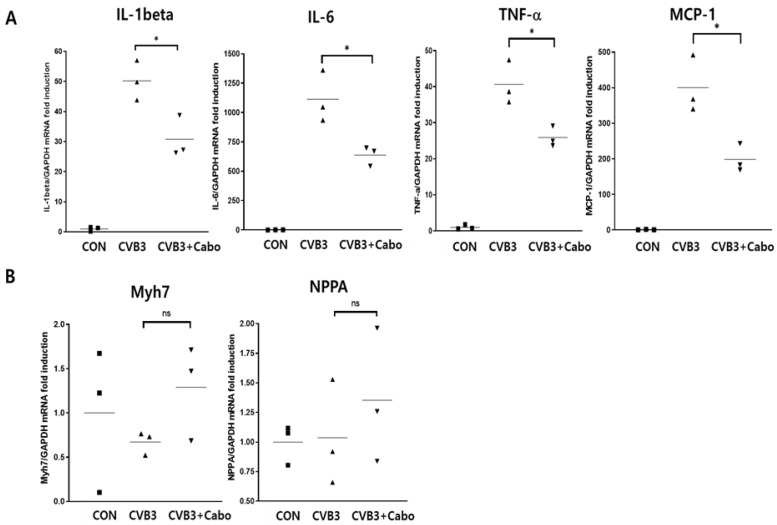
Quantitative RT-PCR measured myocardium damage and inflammatory cytokine production. (**A**) RNA was extracted from the hearts of each group of mice (n = 3 in each group). Heart damage markers (Myh7 and NPPA) and inflammation markers (IL-1beta, IL-6, TNF-alpha, and MCP-1) were quantified. (**B**) The inflammatory cell type was defined. T cell marker (CD4 and CD8), and macrophage marker (CD68) mRNA levels were observed by quantitative RT-PCR. Data are presented as mean ± SD from repeat experiments. CON ■, CVB3 ▲, CVB3+Cabo ▼. ns > 0.05, * *p* < 0.05 in two-tailed Student’s *t*-test.

Heart inflammation was observed by hematoxylin and eosin (H&E) staining on days 3 and 14 post1infection. NIH-ImageJ1.45s software quantified the focal inflammation area in two or three fields of each heart. The inflammation percentage area was dramatically decreased by caboxamycin treatment compared to the CVB3 group (Figure 5A,B). CVB3 infection activates innate immunity and enhances inflammatory cell infiltration into the virus-infected damaged myocytes [27,28]. Heart inflammatory cytokine mRNA expression was observed by quantitative RT-PCR. IL-1beta, IL-6, TNF-alpha, and MCP-1 were significantly decreased in the hearts of caboxamycin-treated mice. In addition, the myocardium was protected against damage (Figure 6A). However, T-lymphocyte and macrophage immune cell populations were not different between the two groups (Figure 6B). These results demonstrated that the inhibition of virus replication by caboxamycin treatment was effective in reducing inflammatory cell infiltration and cytokine production in the hearts of CVB3-infected mice. However, myocardium damage had not yet occurred at the early stage of CVB3 infection.

## 4. Discussion

Enterovirus infection can trigger severe arrhythmias and the development of chronic myocarditis and heart failure [1,2,3,4]. It is the cause of central nervous system (CNS) infection in children. Hand, foot, and mouth disease caused by enterovirus71 (EV71) can cause aseptic meningitis and brain stem encephalitis with respiratory failure and is associated with high mortality [8,9,29]. Coxsackievirus B3 (CVB3) has a gene structure and life history that are comparable to those of known enteroviruses, and the same vaccine development methods have been used for these two groups. However, no effective enterovirus vaccines have been developed. In this study, we generated a new antiviral agent from purified compounds identical to those found in a natural ocean microorganism. These compounds are structurally similar to those found in nature, yet when introduced into the body, their actions may differ. It is also unclear whether natural compounds play a role in the body. Nonetheless, pure natural compounds are expected to be substantially less cytotoxic when absorbed into the body than other synthetic compounds. To define the antiviral role of caboxamycin, we administered caboxamycin in a CVB3-induced myocarditis mouse model.

CVB3 infection is the main cause of myocarditis and cardiac muscle damage [30]. The protective effect of caboxamycin against CVB3 infection was tested in HeLa cells and in Balb/C mice. The result showed that co-treatment with caboxamycin significantly suppressed CVB3 infection and cell mortality. In addition, caboxamycin reduced apoptosis and damage to HeLa cells by inhibiting virus replication and capsid protein (VP1) production. The cleavage of eIF4G1, a transcription initiation factor cleaved by viral protease 2A [31], was notably reduced under caboxamycin treatment. However, interestingly, 1 h after administration, caboxamycin did not show a dramatic antiviral effect. This may mean that the drug’s antiviral effect is dependent on treatment timing [32]. CVB3 is a positive single-strand RNA virus, and producing negative strand RNA is crucial for viral genome amplification. Positive single-strand RNA viruses are a popular research subject nowadays due to the impact of the SARS-CoV2 pandemic. These viruses have very short infection times and, in a few hours, amplify their genomes and proteins. Therefore, they cause very acute, severe damage to infected organisms. Previous reports showed that enterovirus replication occurs by activating ERK, a cell signaling molecule [12]. MAPK (mitogen-activated protein kinases) p38 activity was significantly suppressed following the administration of the drug, and the cells remained viable. This is a very important finding which suggests that caboxamycin may be applied for other antiviral or anticancer therapies. Marine-derived actinomycetes have been considered an essential source of structurally distinct secondary metabolites with various biological activities, including antiviral, anti-tumor, antimicrobial, and anticancer properties [22]. Caboxamycin (1), a benzoxazole class of natural products, was first isolated from the marine bacterium *Streptomyces* sp., SC0774. Here, caboxamycin (1) was administered in a CVB3-induced myocarditis mouse model. The treatment dramatically maintained mouse survival while decreasing heart inflammation and virus replication.

In conclusion, caboxamycin (1) inhibited apoptosis and virus replication in CVB3-infected cells. Inflammatory cell infiltration and inflammatory cytokine expression, associated with CVB3 infection, were dramatically decreased in the hearts of CVB3-induced myocarditis mice. In summary, caboxamycin (1) could be used for the development of a new therapeutic drug for CVB3 treatment.

## Figures and Tables

**Table 1:** Real-time PCR primer sequences.

Gene Name	Sense (5′ → 3′)	Antisense (5′ → 3′)
IL-1β	TTG ACG GAC CCC AAA GAG TG	ACT CCT GTA CTC GTG GAA GA
IL-6	GTA CTC CAG AAG ACC AGA GG	TGC TGG TGA CAA CCA CGG CC
TNF-α	TTG ACC TCA GCG CTG AGT TG	CCT GTA GCC CAC GTC GTA GC
MCP-1	ACC TGG ATC GGA ACC AAA TG	CCT TAG GGC AGA TGC AGT TTT AA
NPPA	TACAGTGCGGTGTCCAACACAG	TGCTTCCTCAGTCTGCTCACTC
Myh7	GCTGAAAGCAGAAAGAGATTATC	TGGAGTTCTTCTCTTCTGGAG
GAPDH	ATC AAC GAC CCC TTC ATT GAC C	CCA GTA GAC TCC ACG ACA TAC TCA GC

## Data Availability

The data presented in this study are available on request from the corresponding author.

## References

[1] Huber S.A., Lodge P.A. (1986). Coxsackievirus B-3 myocarditis. Identification of different pathogenic mechanisms in DBA/2 and Balb/c mice. Am. J. Pathol..

[2] Feldman A.M., McNamara D. (2000). Myocarditis. N. Engl. J. Med..

[3] Woodruff J.F. (1980). Viral myocarditis. A review. Am. J. Pathol..

[4] Knowlton K.U. (2017). Myocarditis: An Intersection Between Genetic and Acquired Causes of Human Cardiomyopathy. J. Am. Coll. Cardiol..

[5] Liu B.M., Mulkey S.B., Campos J.M., DeBiasi R.L. (2024). Laboratory diagnosis of CNS infections in children due to emerging and re-emerging neurotropic viruses. Pediatr. Res..

[6] Abzug M.J., Cloud G., Bradley J., Sanchez P.J., Romero J., Powell D., Lepow M., Mani C., Capparelli E.V., Blount S. (2003). Double blind placebo-controlled trial of pleconaril in infants with enterovirus meningitis. Pediatr. Infect. Dis. J..

[7] Setia A., Bhatia J., Bhattacharya S. (2022). An Overview of Acute Flaccid Myelitis. CNS Neurol. Disord. Drug Targets.

[8] Nayak G., Bhuyan S.K., Bhuyan R., Sahu A., Kar D., Kuanar A. (2022). Global emergence of Enterovirus 71: A systematic review. Beni-Suef Univ. J. Basic Appl. Sci..

[9] Lu G., Qi J., Chen Z., Xu X., Gao F., Lin D., Qian W., Liu H., Jiang H., Yan J. (2011). Enterovirus 71 and coxsackievirus A16 3C proteases: Binding to rupintrivir and their substrates and anti-hand, foot, and mouth disease virus drug design. J. Virol..

[10] Zhao Z., Cai T.Z., Lu Y., Liu W.J., Cheng M.L., Ji Y.Q. (2015). Coxsackievirus B3 induces viral myocarditis by upregulating toll-like receptor 4 expression. Biochemistry.

[11] Herskowitz A., Beisel K.W., Wolfgram L.J., Rose N.R. (1985). Coxsackievirus B3 murine myocarditis: Wide pathologic spectrum in genetically defined inbred strains. Hum. Pathol..

[12] Xiong D., Yajima T., Lim B.K., Stenbit A., Dublin A., Dalton N.D., Summers-Torres D., Molkentin J.D., Duplain H., Wessely R. (2007). Inducible cardiac-restricted expression of enteroviral protease 2A is sufficient to induce dilated cardiomyopathy. Circulation.

[13] Lim B.K., Peter A.K., Xiong D., Narezkina A., Yung A., Dalton N.D., Hwang K.K., Yajima T., Chen J., Knowlton K.U. (2013). Inhibition of Coxsackievirus-associated dystrophin cleavage prevents cardiomyopathy. J. Clin. Investig..

[14] Yun S.H., Lee W.G., Kim Y.C., Ju E.S., Lim B.K., Choi J.O., Kim D.K., Jeon E.S. (2012). Antiviral activity of coxsackievirus B3 3C protease inhibitor in experimental murine myocarditis. J. Infect. Dis..

[15] Lin L.T., Hsu W.C., Lin C.C. (2014). Antiviral natural products and herbal medicines. J. Tradit. Complement. Med..

[16] Opavsky M.A., Martino T., Rabinovitch M., Penninger J., Richardson C., Petric M., Trinidad C., Butcher L., Chan J., Liu P.P. (2002). Enhanced ERK-1/2 activation in mice susceptible to coxsackievirus-induced myocarditis. J. Clin. Investig..

[17] Nagoshi T., Matsui T., Aoyama T., Leri A., Anversa P., Li L., Ogawa W., del Monte F., Gwathmey J.K., Grazette L. (2005). PI3K rescues the detrimental effects of chronic Akt activation in the heart during ischemia/reperfusion injury. J. Clin. Investig..

[18] Wang S., Zhu X., Xiong L., Ren J. (2017). Ablation of Akt2 prevents paraquat-induced myocardial mitochondrial injury and contractile dysfunction: Role of Nrf2. Toxicol. Lett..

[19] Lee Y.G., Park J.H., Jeon E.S., Kim J.H., Lim B.K. (2016). Fructus Amomi Cardamomi Extract Inhibit Coxsackievirus-B3 Induced Myocarditis in Murine Myocarditis Model. J. Microbiol. Biotechnol..

[20] Fenical W., Jensen P.R. (2006). Developing a new resource for drug discovery: Marine actinomycete bacteria. Nat. Chem. Biol..

[21] Lam K.S. (2006). Discovery of novel metabolites from marine actinomycetes. Curr. Opin. Microbiol..

[22] Losada A.A., Cano-Prieto C., Garcia-Salcedo R., Brana A.F., Mendez C., Salas J.A., Olano C. (2017). Caboxamycin biosynthesis pathway and identification of novel benzoxazoles produced by cross-talk in Streptomyces sp. NTK 937. Microb. Biotechnol..

[23] Hohmann C., Schneider K., Bruntner C., Irran E., Nicholson G., Bull A.T., Jones A.L., Brown R., Stach J.E., Goodfellow M. (2009). Caboxamycin, a new antibiotic of the benzoxazole family produced by the deep-sea strain Streptomyces sp. NTK 937. J. Antibiot..

[24] Xiong D., Lee G.H., Badorff C., Dorner A., Lee S., Wolf P., Knowlton K.U. (2002). Dystrophin deficiency markedly increases enterovirus-induced cardiomyopathy: A genetic predisposition to viral heart disease. Nat. Med..

[25] Knowlton K.U., Jeon E.S., Berkley N., Wessely R., Huber S. (1996). A mutation in the puff region of VP2 attenuates the myocarditic phenotype of an infectious cDNA of the Woodruff variant of coxsackievirus B3. J. Virol..

[26] Shin H.H., Jeon E.S., Lim B.K. (2023). Macrophage-Specific Coxsackievirus and Adenovirus Receptor Deletion Enhances Macrophage M1 Polarity in CVB3-Induced Myocarditis. Int. J. Mol. Sci..

[27] Cooper L.T. (2009). Myocarditis. N. Engl. J. Med..

[28] Fairweather D., Yusung S., Frisancho S., Barrett M., Gatewood S., Steele R., Rose N.R. (2003). IL-12 receptor beta 1 and Toll-like receptor 4 increase IL-1 beta- and IL-18-associated myocarditis and coxsackievirus replication. J. Immunol..

[29] Chen Y.C., Yu C.K., Wang Y.F., Liu C.C., Su I.J., Lei H.Y. (2004). A murine oral enterovirus 71 infection model with central nervous system involvement. J. Gen. Virol..

[30] Klingel K., Kandolf R. (1993). The role of enterovirus replication in the development of acute and chronic heart muscle disease in different immunocompetent mouse strains. Scand. J. Infect. Dis. Suppl..

[31] Castello A., Alvarez E., Carrasco L. (2006). Differential cleavage of eIF4GI and eIF4GII in mammalian cells. Effects on translation. J. Biol. Chem..

[32] Mohamud Y., Fu C., Fan Y.M., Zhang Y.L., Lin J.F.C., Hwang S.W., Wang Z.C., Luo H. (2024). Activation of cGAS-STING suppresses coxsackievirus replication via interferon-dependent signaling. Antiviral Res..

